# Supplementary material for: Metabolic benefits of 17α-estradiol in liver are partially mediated by ERβ in male mice
Source: Sci Rep. 2023 Jun 17;13:9841. doi: 10.1038/s41598-023-37007-1 (PMC10276872; doi:10.1038/s41598-023-37007-1)
Supplement: Supplementary file 1 — Supplementary Figures. [file 41598_2023_37007_MOESM1_ESM.pdf]

SUPPLEMENTARY FIGURES AND LEGENDS

Supplemental Figure 1

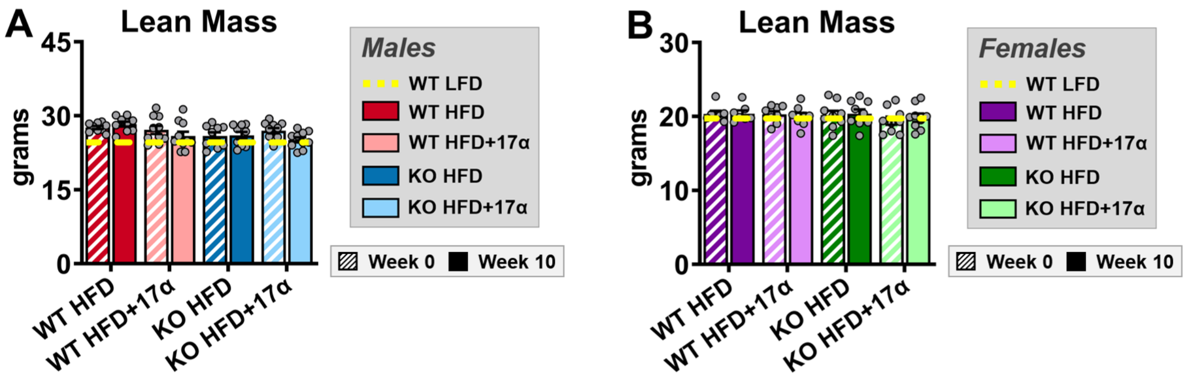

Supplemental Figure 1. Neither ERβ ablation, nor 17α-E2 treatment, affects lean mass in obese mice of either sex. (A) Lean mass at baseline (week 0; striped) and week 10 (solid) in male WT and ERβKO mice [n=9-10/group/timepoint]. (B) Lean mass at baseline (week 0; striped) and week 10 (solid) in female WT and ERβKO mice [n=7-9/group/timepoint]. Age-matched, WT, LFD-fed mice were also evaluated as a normal-weight reference group and their corresponding means for both sexes are depicted as dashed yellow lines [n=9/group/timepoint]. All data are presented as mean ± SEM and were analyzed within sex by two-way repeated measures ANOVA with Tukey post-hoc comparisons.

Supplemental Figure 2

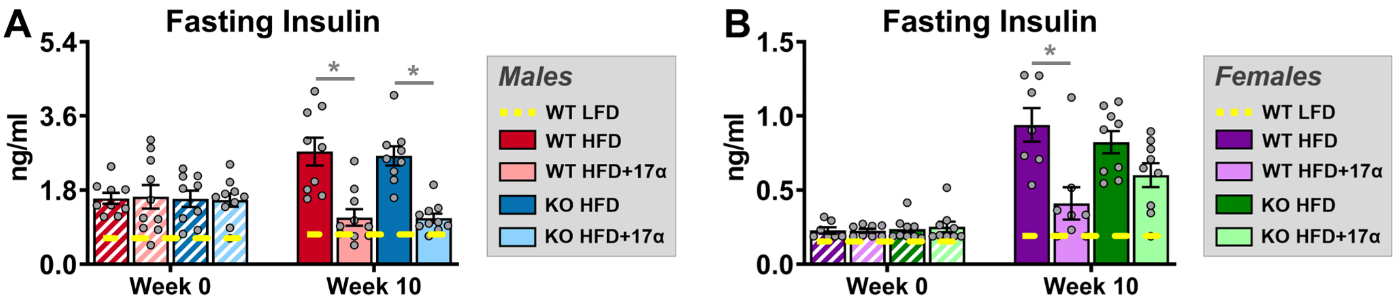

Supplemental Figure 2. ERβ partially mediates 17α-E2 effects on fasting insulin in obese female, but not male, mice. (A) Fasting insulin at baseline (week 0; striped) and week 10 (solid) in male WT and ERβKO mice [n=9-10/group/timepoint]. (B) Fasting insulin at baseline (week 0; striped) and week 10 (solid) in female WT and ERβKO mice [n=7-9/group/timepoint]. Age-matched, WT, LFD-fed mice were also evaluated as a normal-weight reference group and their corresponding means for both sexes are depicted as dashed yellow lines [n=9/group/timepoint]. All data are presented as mean ± SEM and were analyzed within sex by two-way repeated measures ANOVA with Tukey post-hoc comparisons. * represents differences within genotypes across treatment groups at each timepoint. *$p < 0.05$. We did not indicate statistical differences between week 0 and week 10 for purposes of visual clarity.

Supplemental Figure 3

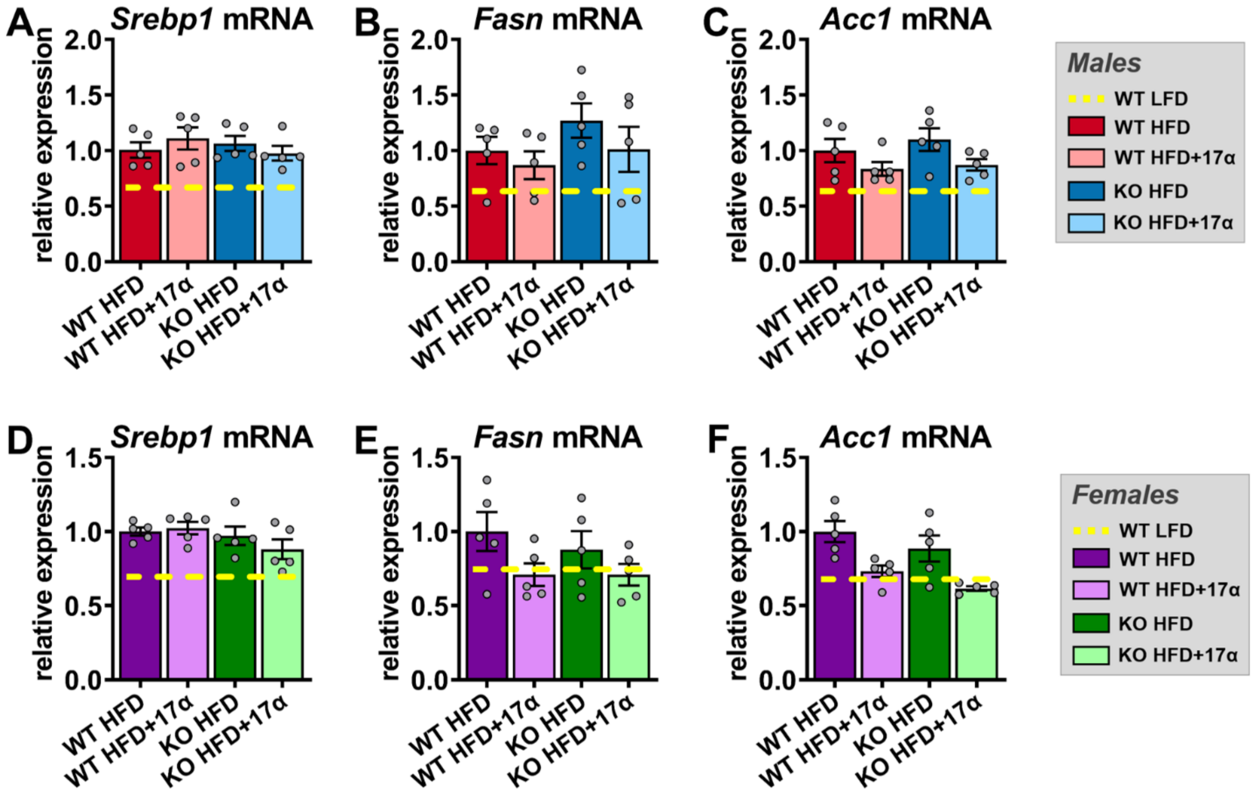

Supplemental Figure 3. Neither ERβ ablation, nor 17α-E2 treatment, effects hepatic transcriptional markers of lipogenesis in obese mice of either sex. (A) *Srebp1* mRNA [n=5/group], (B) *Fasn* mRNA [n=5/group], and (C) *Acc1* mRNA [n=5/group] in liver from male WT and ERβKO mice. (D) *Srebp1* mRNA [n=5/group], (E) *Fasn* mRNA [n=5/group], and (F) *Acc1* mRNA [n=5/group] in liver from female WT and ERβKO mice. Age-matched, WT, LFD-fed mice were also evaluated as a normal-weight reference group and their corresponding means for both sexes are depicted as dashed yellow lines [n=5/group]. All data are presented as mean ± SEM and were analyzed within sex by two-way ANOVA with Tukey post-hoc comparisons.

Supplemental Figure 4

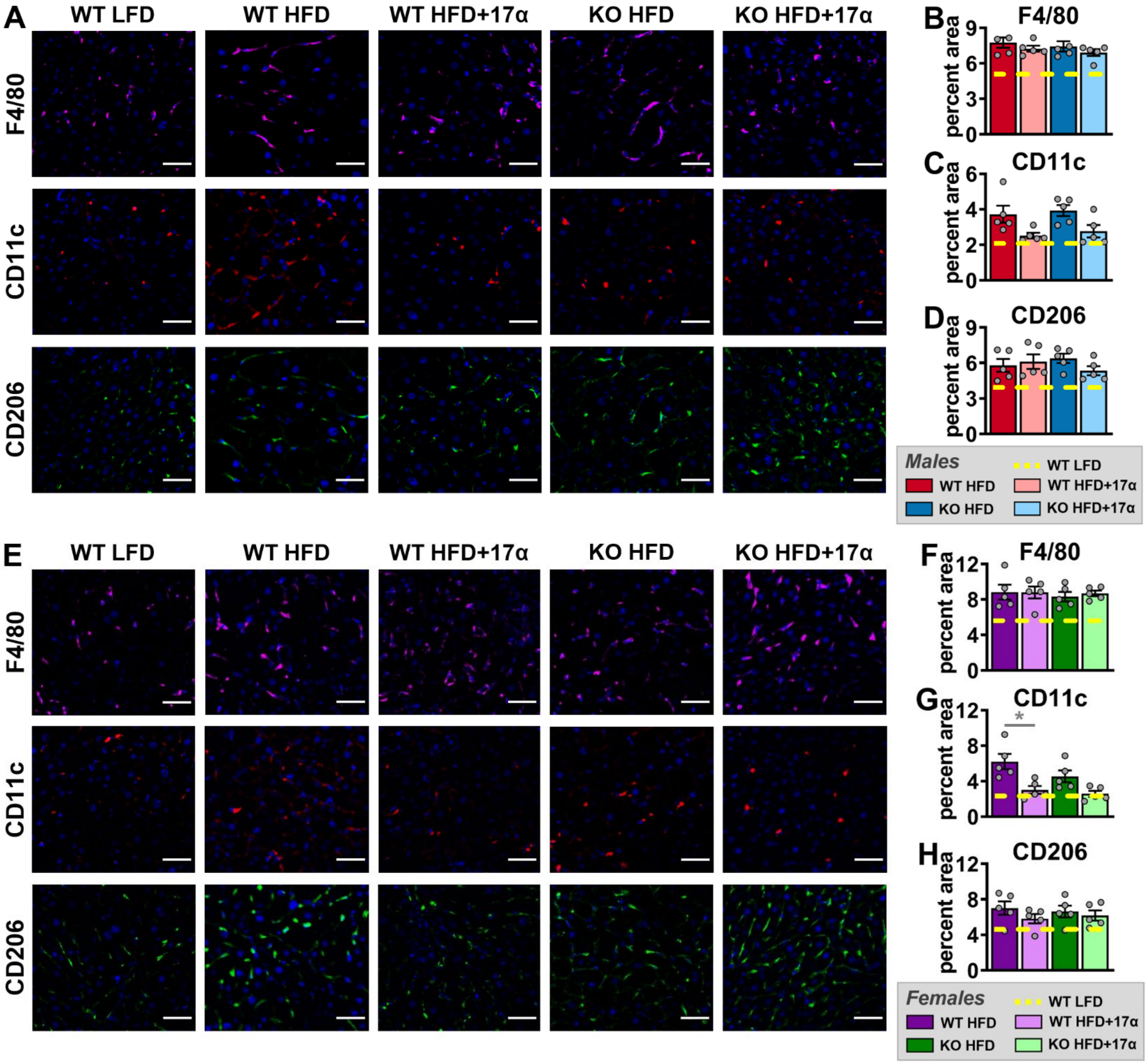

Supplemental Figure 4. 17α-E2 attenuates proinflammatory macrophage responses in female, but not male, mice in a ERβ-dependent manner. (A) Representative immunofluorescence images of F4/80 (total macrophages), CD11c (M1, pro-inflammatory macrophages), and CD206 (M2, anti-inflammatory macrophages) in liver from male WT and ERβKO mice (magnification = 320X; scale bar = 50 μm). (B) Percent area for F4/80 [n=5/group], (C) Percent area for CD11c [n=5/group], and (D) Percent area for CD206 [n=5/group] in liver from male WT and ERβKO mice. (E) Representative immunofluorescence images of F4/80 (total macrophages), CD11c (M1, pro-inflammatory macrophages), and CD206 (M2, anti-inflammatory macrophages) in liver from female WT and ERβKO mice (magnification = 320X; scale bar = 50 μm). (F) Percent area for F4/80 [n=5/group], (G) Percent area for CD11c [n=5/group], and (H) Percent area for CD206 [n=5/group] in liver from female WT and ERβKO mice. Age-matched, WT, LFD-fed mice were also evaluated as a normal-weight reference group and their corresponding means for both sexes are depicted as dashed yellow lines [n=5/group]. All data are presented as mean ± SEM and were analyzed within sex by two-way ANOVA with Tukey post-hoc comparisons. * represents differences within genotypes across treatment groups. *$p < 0.05$.

Supplemental Figure 5

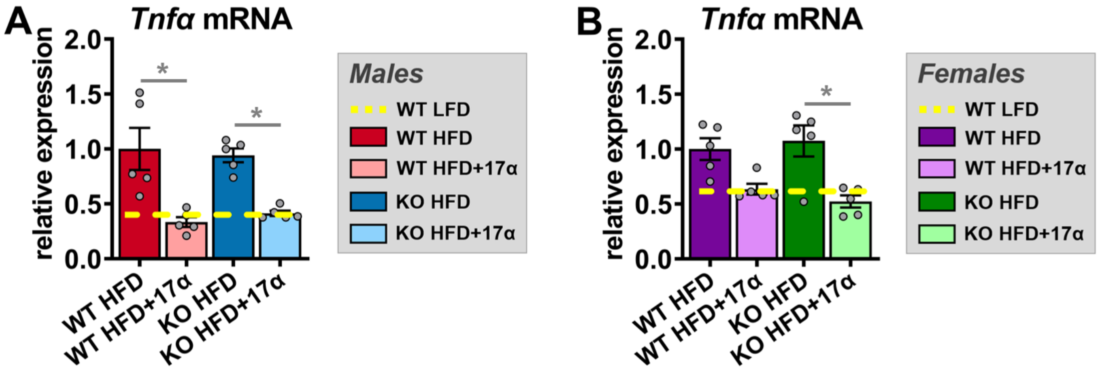

Supplemental Figure 5. 17α-E2 suppresses hepatic tumor necrosis factor α transcripts in both sexes in an ERβ-independent manner. (A) *TNFα* mRNA in liver from male WT and ERβKO mice [n=5/group]. (B) *TNFα* mRNA in liver from female WT and ERβKO mice [n=5/group]. Age-matched, WT, LFD-fed mice were also evaluated as a normal-weight reference group and their corresponding means for both sexes are depicted as dashed yellow lines [n=5/group]. All data are presented as mean ± SEM and were analyzed within sex by two-way ANOVA with Tukey post-hoc comparisons. * represents differences within genotypes across treatment groups. *$p < 0.05$.
