# Supplementary material for: Metabolic benefits of 17α-estradiol in liver are partially mediated by ERβ in male mice
Source: Sci Rep. 2023 Jun 17;13:9841. doi: 10.1038/s41598-023-37007-1 (PMC10276872; doi:10.1038/s41598-023-37007-1)

Supplementary Information File

Metabolic benefits of 17α-estradiol in liver are partially mediated by ERβ in male mice

Samim Ali Mondal[1,†], Shivani N. Mann[2,†], Carl van der Linden[1], Roshini Sathiaseelan[1,3], Maria Kamal[4], Snehasis Das[5,6], Matthew P. Bubak[1], Sreemathi Logan[7], Benjamin F. Miller[1,8], Michael B. Stout[1,8]*

Fig. 5B – Male SCD1 & GAPDH Blots [GEL #1]

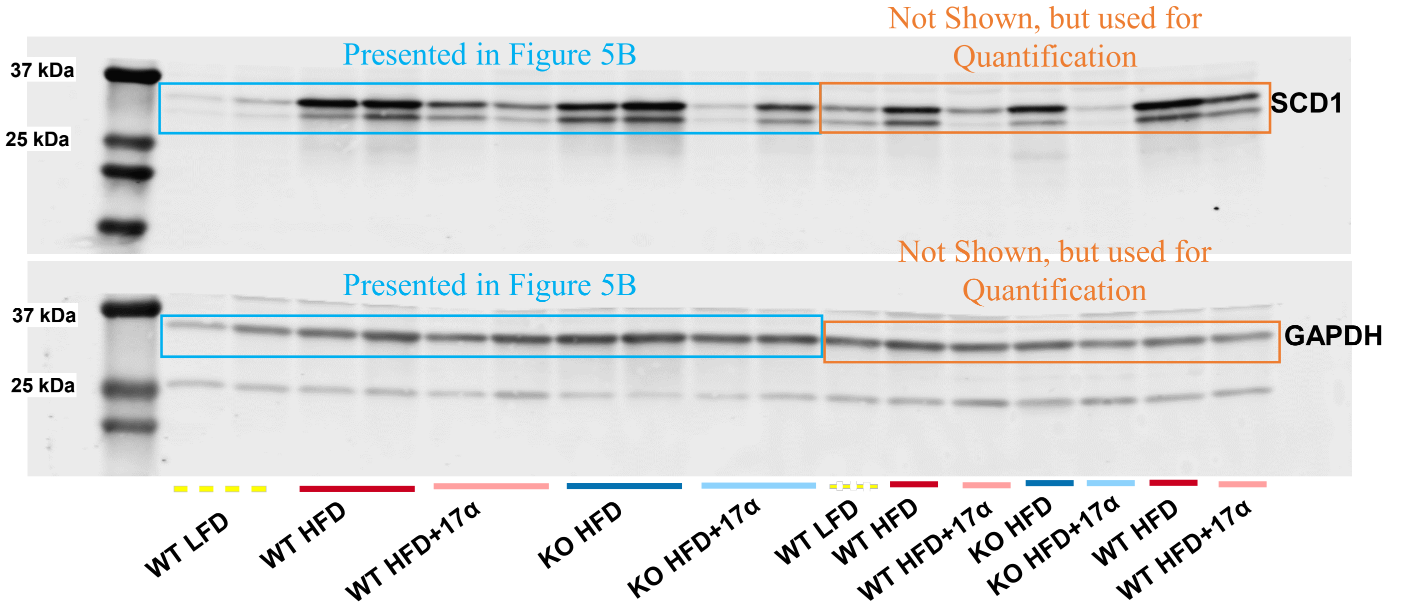

Fig. 5B – Male SCD1 & GAPDH Blots [GEL #2]

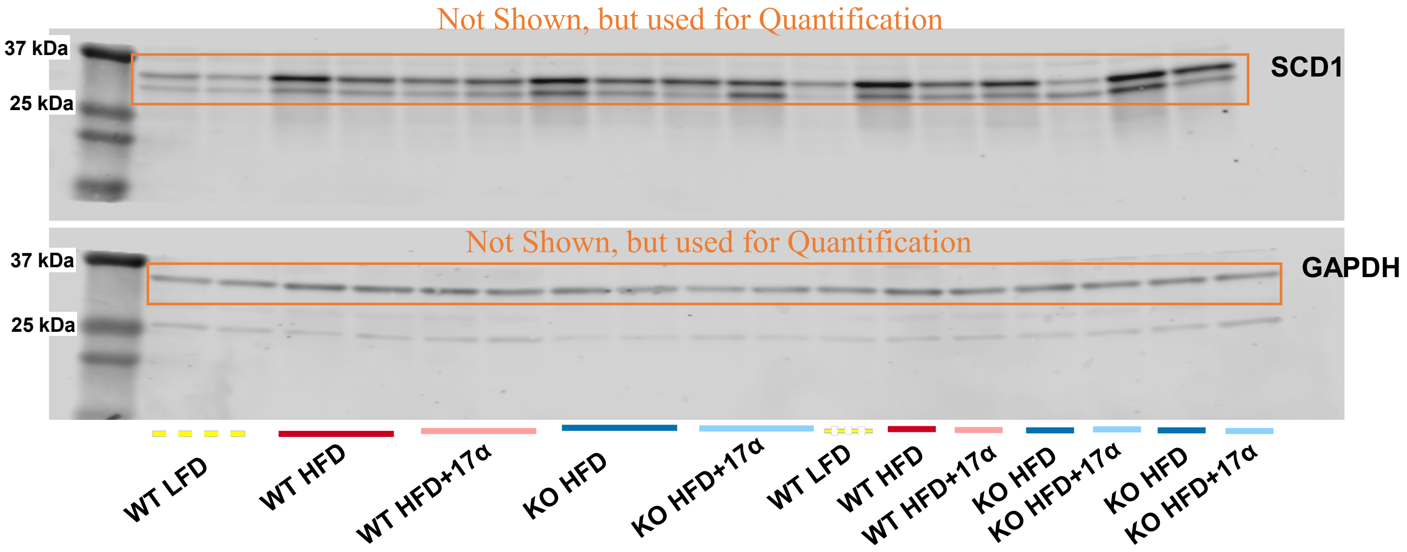

Fig. 5E – Female SCD1 & GAPDH Blots [GEL #1]

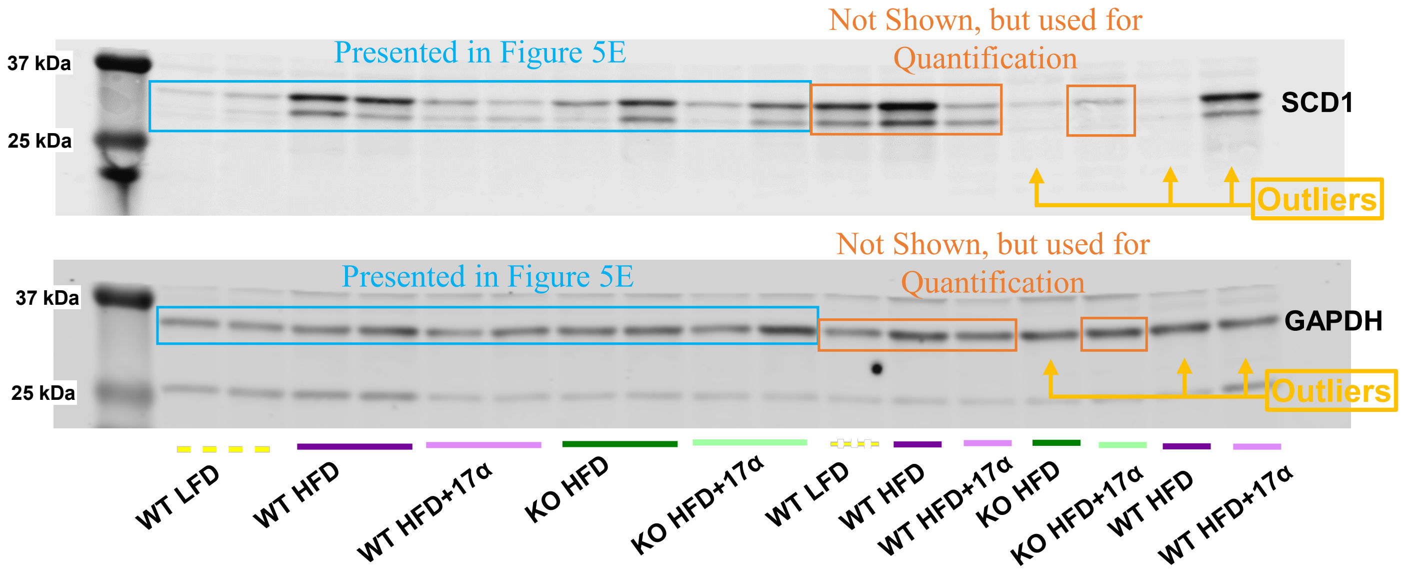

Fig. 5E – Female SCD1 & GAPDH Blots [GEL #2]

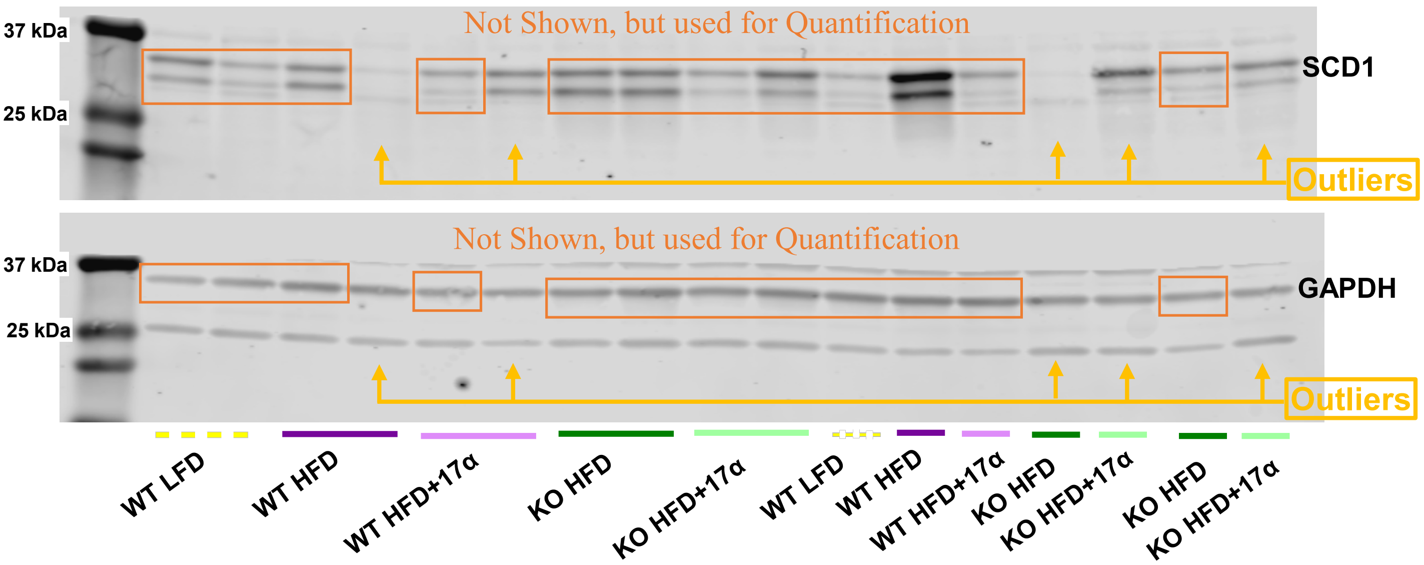

Fig. 6G – HepG2 Cell SCD1 & GAPDH Blots [GEL #1]

Since SCD1 and GAPDH are the same molecular weight we have to run them on separate ProteinSimple Jess Cartridges. Also, since our protein concentrations are fairly low, the images for SCD1 and GAPDH are adjusted to different resolutions for visual purposes. Every treatment group is adjusted to the same resolution.

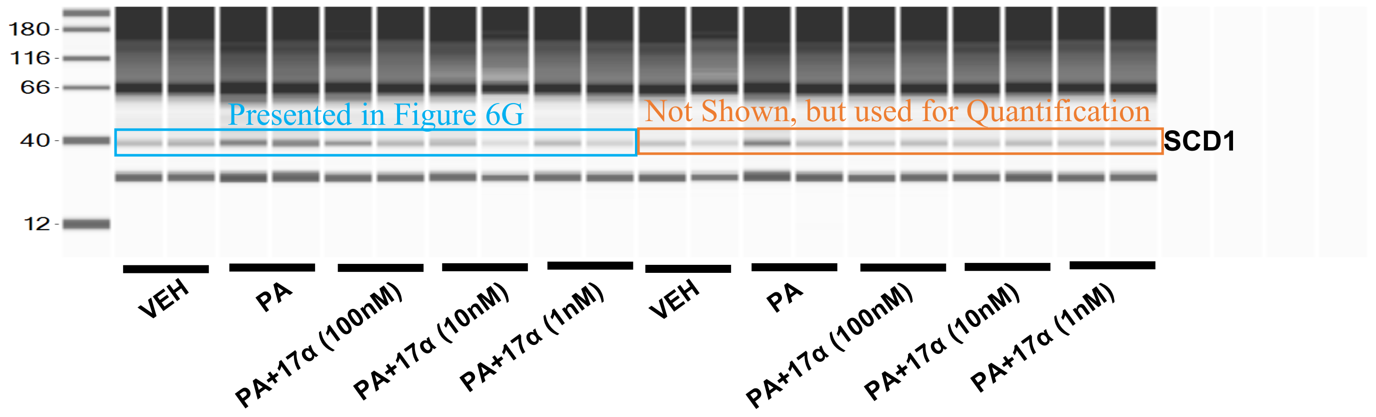

Fig. 6G – HepG2 Cell SCD1 & GAPDH Blots [GEL #2]

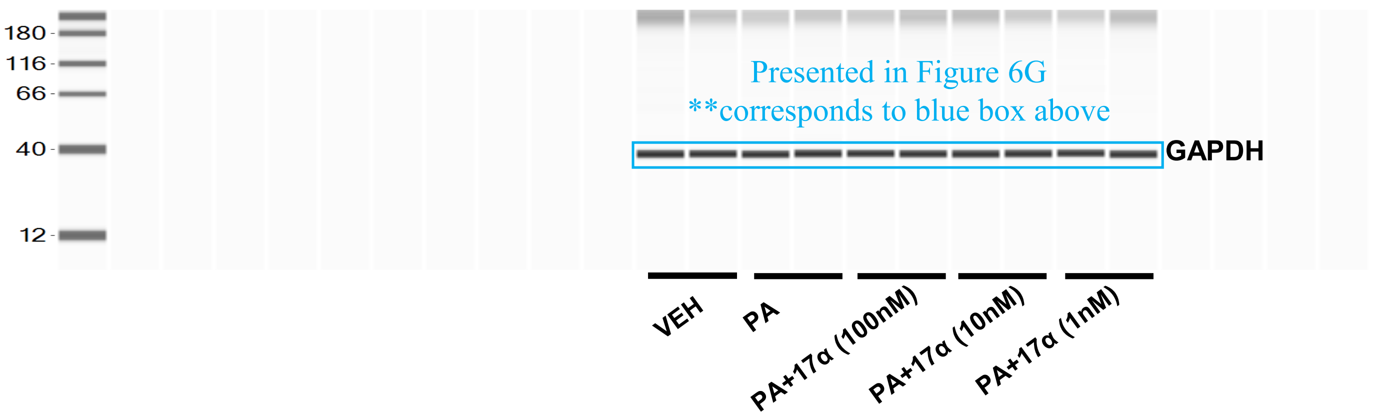

Fig. 6G – HepG2 Cell SCD1 & GAPDH Blots [GEL #3]

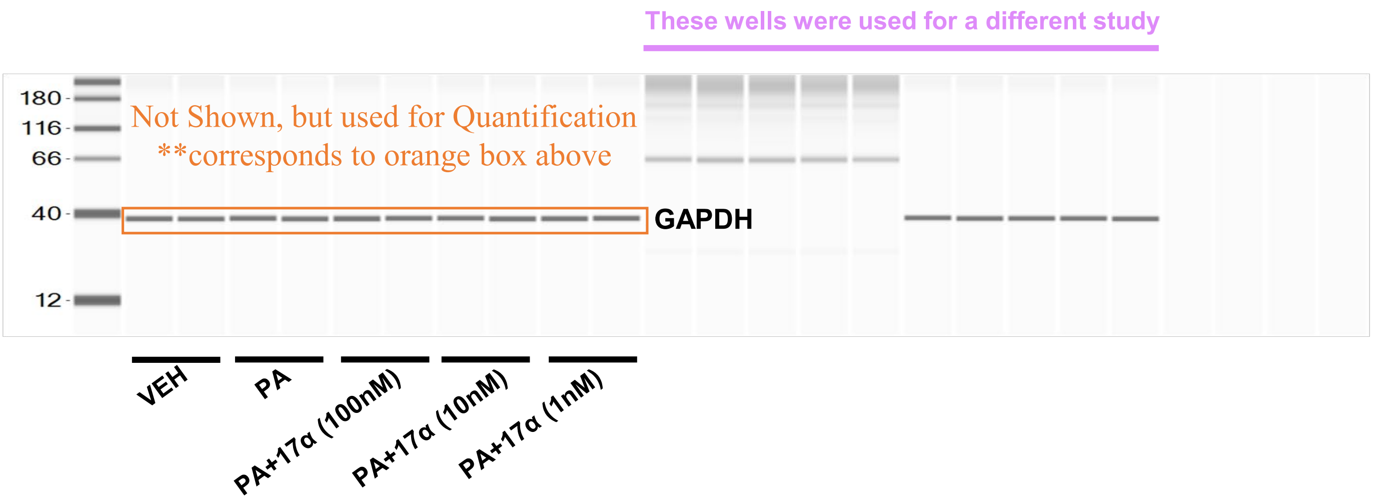

Fig. 6J – LX-2 Cell SCD1 & GAPDH Blots [GEL #1]

Since SCD1 and GAPDH are the same molecular weight we have to run them on separate ProteinSimple Jess Cartridges. Also, since our protein concentrations are fairly low, the images for SCD1 and GAPDH are adjusted to different resolutions for visual purposes. Every treatment group is adjusted to the same resolution.

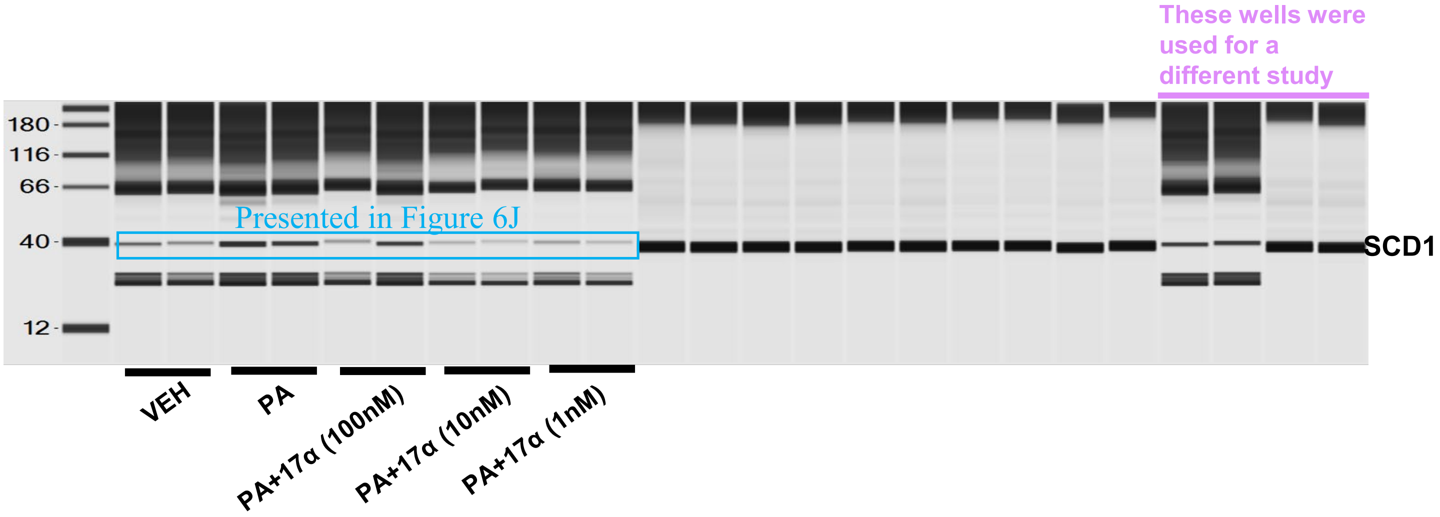

Fig. 6J – LX-2 Cell SCD1 & GAPDH Blots [GEL #1: same gel as above, different intensities]

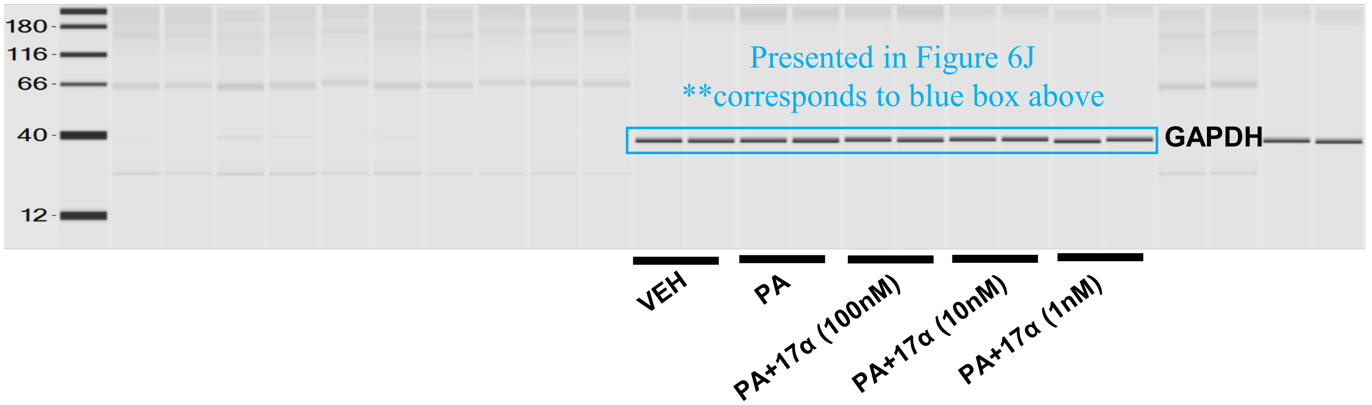

Fig. 6J – LX-2 Cell SCD1 & GAPDH Blots [GEL #2]

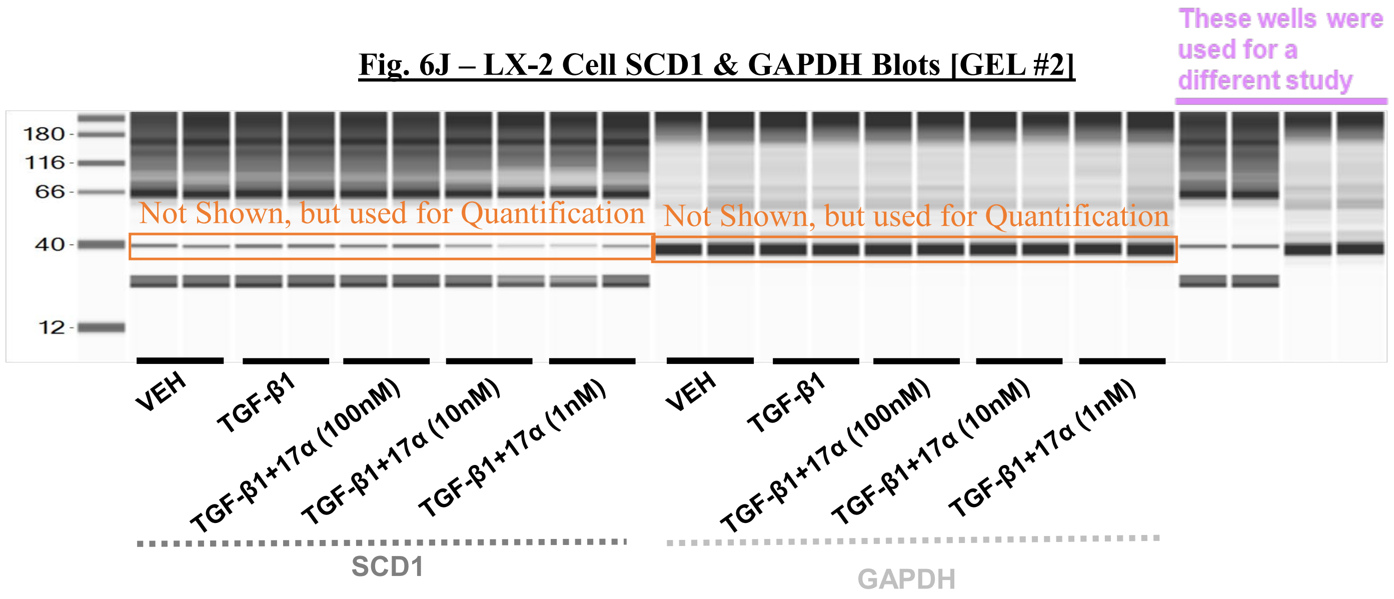

Supplement: Supplementary file 2 — Supplementary Information. [file 41598_2023_37007_MOESM2_ESM.pdf]
